# CT Imaging Patterns in Major Histological Types of Lung Cancer

**DOI:** 10.3390/life14040462

**Published:** 2024-04-01

**Authors:** Cristina Mihaela Ciofiac, Mădălin Mămuleanu, Lucian Mihai Florescu, Ioana Andreea Gheonea

**Affiliations:** 1 Doctoral School, University of Medicine and Pharmacy of Craiova, 200349 Craiova, Romania; cristina.ciofiac@umfcv.ro; 2 Department of Automatic Control and Electronics, University of Craiova, 200585 Craiova, Romania; 3 Department of Radiology and Medical Imaging, University of Medicine and Pharmacy of Craiova, 200349 Craiova, Romania; lucian.florescu@umfcv.ro (L.M.F.); ioana.gheonea@umfcv.ro (I.A.G.)

**Keywords:** lung cancer, adenocarcinoma, squamous cell carcinoma, small cell lung cancer, computed tomography, histopathological examination

## Abstract

Lung cancer ranks as the second most prevalent cancer globally and is the primary contributor to neoplastic-related deaths. The approach to its treatment relies on both tumour staging and histological type determination. Data indicate that the prognosis of lung cancer is strongly linked to its clinical stage, underscoring the importance of early diagnosis in enhancing patient outcomes. Consequently, the choice of an appropriate diagnostic method holds significant importance in elevating both the early detection rate and prognosis of lung cancer. This paper aims to assess computer tomography features specific to the most common lung cancer types (adenocarcinoma, squamous cell carcinomas and small cell lung cancer). Data were collected retrospectively from CT scans of 58 patients pathologically diagnosed with lung cancer. The following CT features were evaluated and recorded for each case: location, margins, structure, lymph node involvement, cavitation, vascular bundle-thickening, bronchial obstruction, and pleural involvement. Squamous cell carcinoma (SQCC) and small cell lung cancer (SCLC) showed a higher incidence of central location, while adenocarcinoma (ADC) showed a significant predilection for a peripheral location. Internal cavitation was mostly observed in SQCC, and a solid structure was observed in almost all cases of ADC. These features can provide information about the prognosis of the patient, considering that NSCLCs are more frequent but tend to demonstrate positive results for targetable driver mutations, such as EGFR, thereby increasing the overall survival. In addition, SCLC presents with early distant spreads, which limits the opportunity to investigate the evolution of tumorigenesis and gene alterations at early stages but can have a rapidly positively response to chemotherapy. The location of the lung cancer exhibits distinct forecasts, with several studies suggesting that peripheral lung tumours offer a more favourable prognosis. Cavity formation appears correlate with a poorer prognosis. Histopathological analysis is the gold standard for diagnosing the type of lung cancer; however, using CT scanning for the purpose of a rough, but fast, preliminary diagnosis has the potential to shorten the waiting time for treatment by helping clinicians and patients to know more about the diagnosis and prognosis.

## 1. Introduction

According to the latest GLOBOCAN estimates, 2,206,771 new cases of lung cancer were diagnosed globally in 2020. Lung cancer is the second most common cancer worldwide, just behind breast cancer, which comprises 11.7% of all cases, while lung cancer comprises 11.4% of all cases. Lung cancer is a significant public health concern, causing a considerable number of deaths globally. It is the leading cause of cancer mortality worldwide, among both sexes, and among men and women separately. In 2020, lung cancer accounted for an estimated 1,796,144 deaths, 18% of all cancer deaths [1]. 

Cigarette smoking is responsible for over 80% of lung cancer cases in the Western world. Globally, tobacco smoking is recognized as the foremost preventable cause of death, primarily owing to the heightened risk of not only lung cancer but also other malignancies such as those affecting the bladder and colorectum [2]. Furthermore, genetic elements also serve crucial functions in the onset of cancer. Research involving twins [3] and estimations of heritability derived from genome-wide association studies (GWASs) [4,5] have revealed that genetic factors play a considerably smaller role in the occurrence of lung cancer compared to environmental factors such as smoking. Zhang et al. 2022 aimed to investigate the risk of lung cancer in people with different genetic risks and smoking habits through a prospective cohort study of 345,794 European ancestry participants followed up for 7.2 [6.5–7.8] years. They concluded that both genetic risk and smoking were independently associated with higher lung cancer risk, but the increased risk of smoking was much more significant than heredity [6]. 

Another systematic review and meta-analysis investigated the sex-specific association between smoking and lung cancer. Data from 29 studies representing 99 cohort studies, 7 million individuals and >50 000 incident lung cancer cases were included. The study concluded that smoking yields similar risks of lung cancer in women compared to men [7].

Other risk factors include exposure to second-hand smoke, occupational hazards (such as asbestos, radon and certain chemicals), air pollution, hereditary cancer syndromes, and previous chronic lung diseases [8]. Efforts aimed at preventing the increasing global prevalence of lung cancer should focus on addressing risk factors, including smoking, occupational and environmental exposures, as well as infections such as HIV and tuberculosis [9]. 

Lung cancer exhibits low survival rates, with a mere 18% 5-year survival rate in the United States. This contrasts with the significantly higher survival rates observed in breast, colon, and prostate cancer, reaching approximately 90%, 65%, and nearly 100%, respectively [10]. The low survival rates are directly associated with the fact that most cases of lung cancer are only detected in advanced stages. In instances where the disease is localized, survival rates can reach as high as 55.2%. However, a mere 16% of patients receive an early-stage diagnosis [11]. This difference in survival rates further highlights the absolute need for a reliable screening tool for patients at risk for lung cancer. 

Imaging is crucial in overseeing cancer patient care. It not only aids in diagnosis by identifying areas of abnormality and guiding biopsies but also plays a critical role in staging and evaluating the extent of the disease, which in turn helps to determine the appropriate treatment. While most patients are diagnosed in advanced stages due to late-stage symptomatology, individuals with early-stage lung cancer can undergo treatment with the potential for a curative outcome. Therefore, the significance of early imaging diagnosis and accurate radiological staging cannot be overstated. While some cases may lack pulmonary symptoms, they may instead manifest extrapulmonary signs, such as liver failure, bone pain, or even soft-tissue metastases [12]. 

Accurate staging of lung cancer patients is vital, as it provides detailed insights into the extent of the disease, both locally and distantly. This information not only guides the selection of appropriate treatment but also helps in estimating the prognosis. Various staging methods are employed, including advanced imaging techniques such as computed tomography (CT), 2-deoxy-2-(18F) fluoro-d-glucose positron emission tomography (FDG-PET), and integrated FDG-PET/CT scans. Additionally, needle-based biopsy procedures, like endobronchial ultrasound transbronchial needle aspiration (EBUS-TBNA) or oesophageal ultrasound needle aspiration (EUS-NA), transbronchial needle aspiration (TBNA), narrow-band imaging bronchoscopy/endoscopy (NBI), and CT-guided transthoracic fine needle aspiration (TTFNA), are utilized. Surgical techniques, such as mediastinoscopy, mediastinotomy, or video-assisted thoracoscopic (VATS) procedures, also play a crucial role in staging [13,14,15,16,17,18].

In day-by-day practice, the initial step in investigating a suspected case of lung cancer mostly still involves a chest radiograph. While it serves as a valuable tool for obtaining preliminary information about the disease, its capabilities are insufficient for comprehensive characterization and staging [19]. The gold standard of lung cancer imaging, upon which subsequent management decisions rely, remains the CT scan, in which the imaging appearances of the primary tumour can exhibit a diverse scale [20]. Whatever the imaging appearance of the suspected lung cancer, obtaining a tissue diagnosis by performing a bronchoscopy or an image-guided biopsy is necessary.

Lung cancer is primarily categorized into non-small-cell lung cancer (NSCLC) and small-cell lung cancer (SCLC). NSCLC constitutes roughly 85–88% of all lung cancers, while SCLC represents approximately 12–15% of cases. NSCLC is classified into three types according to its characteristics and treatment measures, namely, adenocarcinoma (55%), squamous cell carcinoma (35%), and large cell carcinoma (10%) [21].

The histological characteristics of tumours serve as significant indicators for predicting treatment response and prognosis in lung cancer. While tissue sampling remains the gold standard for histological classification, recent advancements in deep learning for medical image analysis suggest the potential of radiological data to complement histology in delineating disease traits and stratifying risk [22].

In clinical practice, histopathology is of utmost importance for the qualitative diagnosis of such patients. In this sense, light microscopic criteria can be used to distinguish between SCLC and NSCLC based on their morphological differences. Typically, SCLC exhibits a higher ratio of nuclear/cytoplasmic, finely granular nuclear chromatin, absence of nucleoli, and frequently presents with a fusiform shape [23]. At the time of diagnosis, SCLC consistently spreads distantly, which limits the opportunity to investigate the evolution of tumorigenesis and gene alterations at early stages [24]. Due to this early onset of metastasis, only a minority of patients with SCLC are suitable candidates for curative lung resection, necessitating adjuvant platinum-etoposide chemotherapy. As a result, most patients are currently undergoing treatment with chemoradiation, with or without immunotherapy [23]. NSCLC, however, is managed by a combination of surgery and adjuvant therapy with newer treatment opportunities and a much better prognosis. Differential diagnosis between adenocarcinoma (ADC) and squamous cell carcinoma (SQCC) is of clinical significance, likewise. Compared with SQCC, adenocarcinoma is associated with a better prognosis [25].

As previously summarized, treatment is highly dependent on stage and histological subtype. According to the guidelines of the National Comprehensive Cancer Network (NCCN), chemotherapy regimens are different for ADC, SQCC and SCLC. Significant progress in lung cancer research has introduced novel treatments, such as epidermal growth factor receptor (EGFR)-targeted therapies, which are advised as the primary treatment for NSCLC with EGFR mutations, with these mutations predominantly manifesting in ADC. Other new treatments, such as Pemetrexed, is contraindicated in SQCC due to the lack of effectiveness [26]. Patients with limited-stage SCLC are candidates for curative-intent radiation therapy and chemotherapy. Patients with extensive-stage disease are treated with chemotherapy, with radiation (RT) reserved for select candidates and for palliation [27,28].

Typically, standard morphologic criteria can differentiate between subtypes of lung cancers, a process that often takes some time, and at other times may not be available for certain patients who cannot tolerate invasive methods. Serial imaging studies indicate that NSCLC might progress swiftly from presentation to the initiation of treatment [10]. Therefore, it is imperative for clinicians to differentiate between these subtypes efficiently and rapidly. The widely available CT scan, recognized as the most cost-effective and efficient noninvasively diagnostic method for lung cancer, is accessible in most hospitals. If CT scans can offer indications to distinguish between these cancer subtypes, clinicians can promptly furnish patients and their families with more information regarding diagnosis, therapy options, prognosis, and costs. This would facilitate a quicker decision-making process and provide more time for patients and their families to prepare.

Given that treatment relies on both tumour staging and the histological subtype of lung cancer [29], and considering that CT has become the preferred modality for screening and diagnosing lung cancer, some recent studies have attempted to establish correlations between various CT features of lung cancer and specific pathological subtypes.

In this study, we retrospectively analysed three major subtypes of lung cancers, ADC, SQCC and SCLC, from November 2022 to April 2023, to compare different morphologic CT features and the clinical pathology, to try and provide a basis for the CT to distinguish between them.

## 2. Materials and Methods

This study was approved by the local medical ethics committee (No. 84/19.02.2024).

Data were retrospectively collected from 58 patients with lung cancer who underwent CT scans from November 2022 to April 2023 in the Imaging Department of University of Medicine and Pharmacy of Craiova. During this selected period, 76 patients presented with a pulmonary tumour. All the patients underwent histopathological diagnosis, either by transthoracic needle biopsy, in which a needle was inserted through the chest wall via CT, or fluoroscopic guidance or transbronchial biopsy using flexible bronchoscopy via the trans-nasal or trans-oral route. Out of the total number of patients, 26 were diagnosed with lung adenocarcinoma, 22 with squamous cell carcinoma, and 10 with small cell lung cancer. The remaining 18 patients presented with alternative forms of malignant or benign lesions that were not within the scope of our study. In this study, we included patients with lung cancer regardless of their up following treatment plan. Exclusion criteria were other types of lung cancer, synchronic cancers, patients who were previously diagnosed with other cancers, and patients with previous surgical interventions of the lung.

All 58 patients underwent chest plain- and enhanced-CT scans obtained on a single CT systemin helical mode from the apex to the lung base. Technical parameters were as follows: X-ray tube current 200 mA per slice; tube voltage 120 kV; collimation 1.5 mm, pitch 1.5; and field of view (FOV) for small, medium, and large patients. All image data were transmitted directly to our picture-archiving and communication system (PACS). Multi-planar, as well as multiple intensity projection (MIP) reconstruction methods, had been conducted. For this, scans were performed to cover the root of the neck down to the level of the adrenal gland. Iomeron (400 mg/mL) was used to perform the contrast-enhanced scanning. A high-pressure syringe was used to inject the contrast agent (approximately 1–2 mL/kg) from the elbow vein (usually the right elbow vein), with an injection rate of 3.0 mL/s. Thirty millilitres of saline were later injected at the same rate.

Using the blind technique, two radiologists fully revised and studied the CT scan findings, including tumoral features in a particular location (central or peripheral), margin, structure, number, lymph node involvement, the presence or absence of cavitation, signs of vascular bundle thickening, bronchial obstruction, and pleural involvement. One of the radiologists was a senior with 13 years’ experience, the other was a young specialist with 3 years’ experience. Both were blinded from the patients’ pathologic diagnoses.

In addition, the Chi-squared test was used to assess the significance of associations between variables. Specifically, the relationship between each characteristic represented in the figures below and each type of cancer. Each statistical test yielded a corresponding *p*-value, which served as a measure of the strength of association between the variables under investigation. We observed that certain values in our contingency table had low expected counts, which could potentially impact the reliability of the Chi-squared test results, as can be seen in the Section 3.2.4 of the Section 3.

## 3. Results

### 3.1. Patient Characteristics

**Figure 1:**
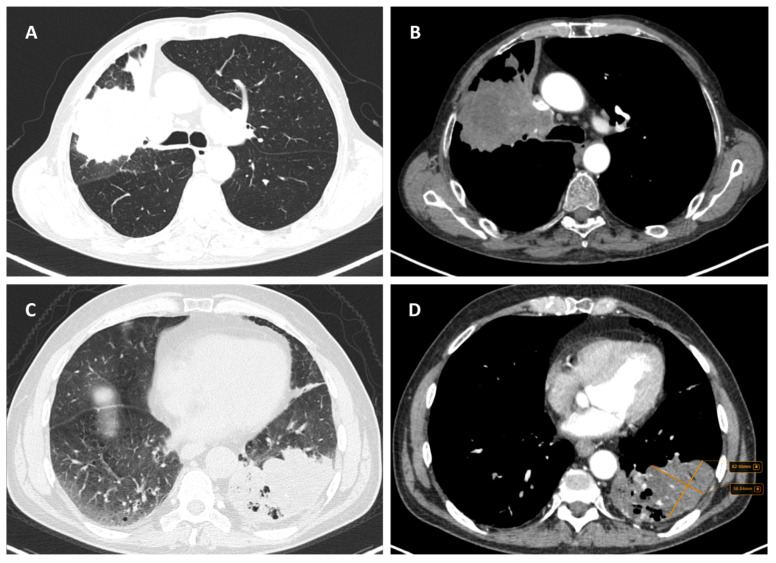
Axial CT of two different cases of adenocarcinoma: (**A**) a right peripheral mass (the maximal tumoral diameter over three centimetres) with spiculated margins, a small atelectatic area and hilar invasion is highlighted on lung window; (**B**) soft-tissue window that shows the same tumour as (**A**) with central necrosis and pleural invasion, including the main right bronchus; and (**C**,**D**) a peripheral mass with adjacent pneumonitis in the left lower lobe.

**Figure 2:**
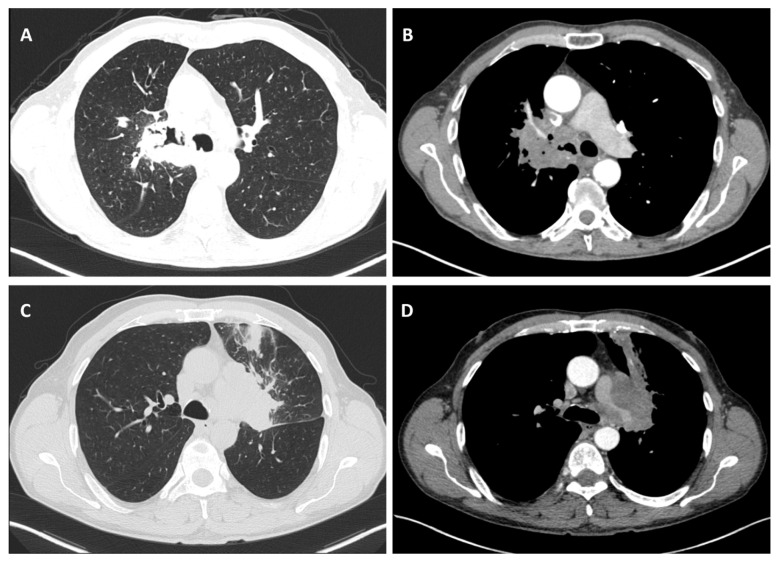
Axial CT of two different cases of squamous cell carcinoma: (**A**,**B**) show a right hilar mass with cavity and air bubbles inside, with invasion of the hilar vessels and right main bronchus extending over less than 2 cm from the carina; and (**C**,**D**) show a left hilar mass with atelectasis, necrosis, septal thickening, and important invasion of the pulmonary trunk and left pulmonary artery.

**Figure 3:**
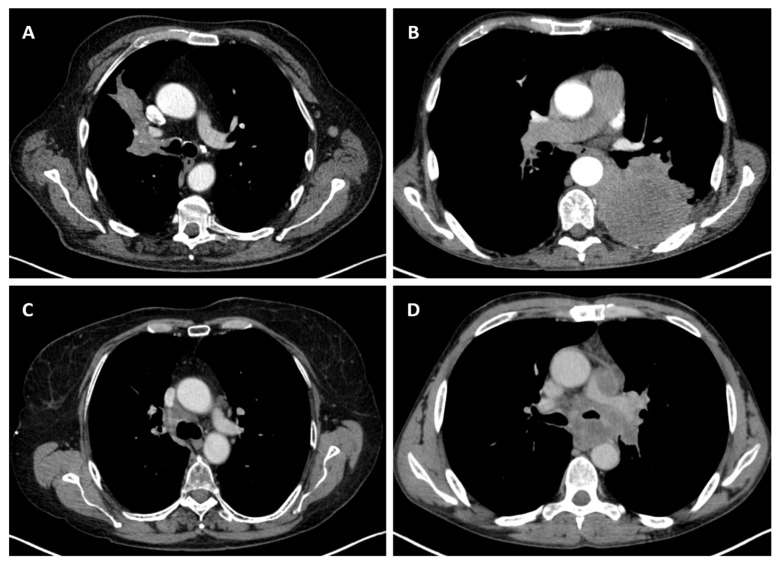
Axial CT of four different cases of small cell lung cancer: (**A**) right hilar mass with segmental atelectasis and expansion into the right main bronchus; (**B**) left voluminous mass with central necrosis, pleural invasion and periaortic extension; (**C**) small-sized central right tumour with paratracheal location; and (**D**) central left tumour with important mediastinal invasion involving the pulmonary trunk, left pulmonary artery, left main bronchus, and oesophagus.

Considering the exclusion criteria previously mentioned, a total of 58 patients were analysed in this study, in which 26 cases were pathologically proven to be adenocarcinoma (Figure 1), 22 cases were shown to be squamous cell carcinoma (Figure 2), and 10 cases were shown to be small cell lung cancer (Figure 3).

Regarding sex distribution, the group included 14 women and 44 males, with ages between 43 and 83 years old. There was no significant difference between the three groups regarding female distribution, with six females in the ADC group, and four females in each of the SQCC and SCLC groups.

The highest prevalence was in the 61–70 age group for all three types of lung cancer, with a higher incidence for the 40–50 age group for small cell lung carcinoma, as compared with ADC and SQCC (Table 1).

### 3.2. Primary Tumour Imaging Findings

#### 3.2.1. Number

**Figure 4:**
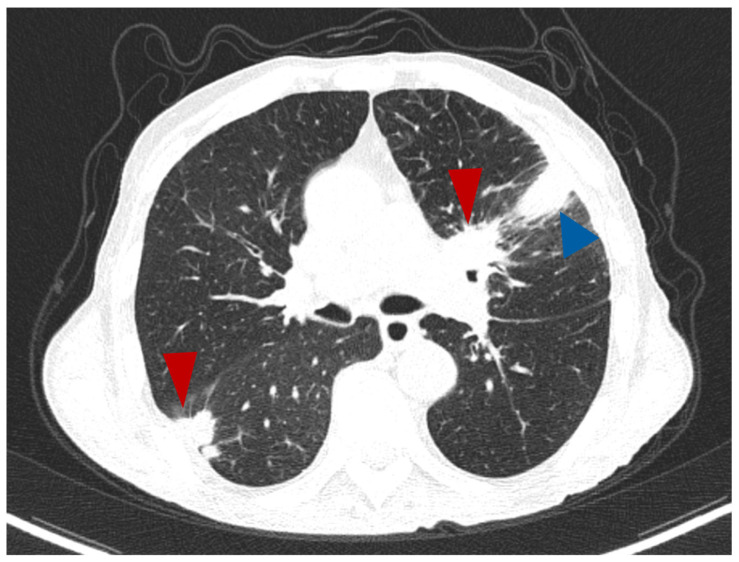
Axial CT lung window—a case of lung ADC with two spiculated masses (red arrowhead), one in the right lower lobe and the other one in upper left lobe, which in this second location combines a segmental atelectasis (blue arrowhead).

A single tumour was observed in almost all cases. There were only two cases with more than one tumoral mass, with CT features suggestive for a primary lesion in the adenocarcinoma group (7.7%), as exemplified in Figure 4, and two cases in the SQCC group (9%). All cases from the SCLC group showed a single primary tumour per patient.

#### 3.2.2. Location

**Figure 5:**
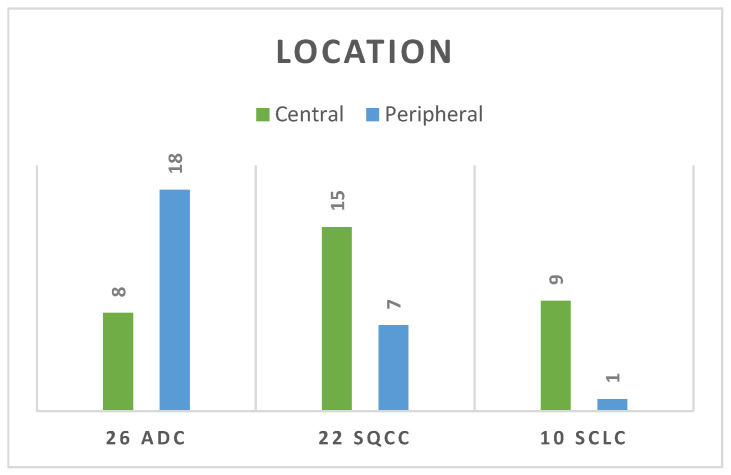
Location of the lesion for each pathological group.

Regarding the location of the lesion, ADC showed a higher incidence of peripheral location (69%) compared with the rest of the lung cancers (30%), with the remainder of the peripheral lesions being SQCC and a single SCLC. SQCC showed a significant predilection for a central location compared with the rest of the lung cancers (*p* = 0.001), as seen in Figure 5.

#### 3.2.3. Tumoral Margins

**Figure 6:**
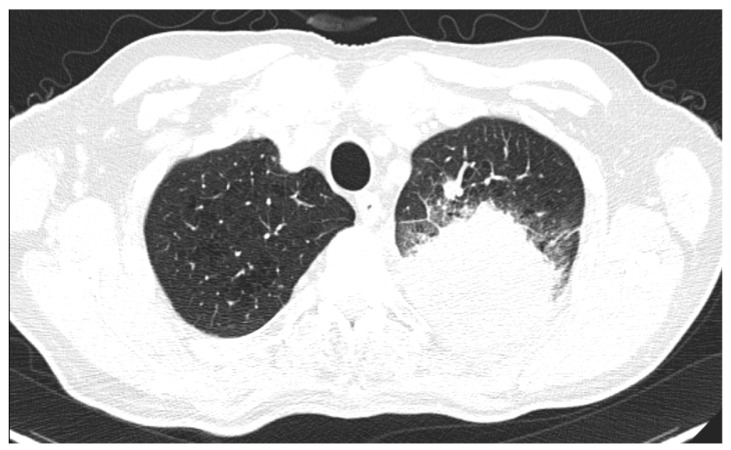
Axial CT lung window view of a spiculated ADC tumour localised in the left pulmonary apex.

**Figure 7:**
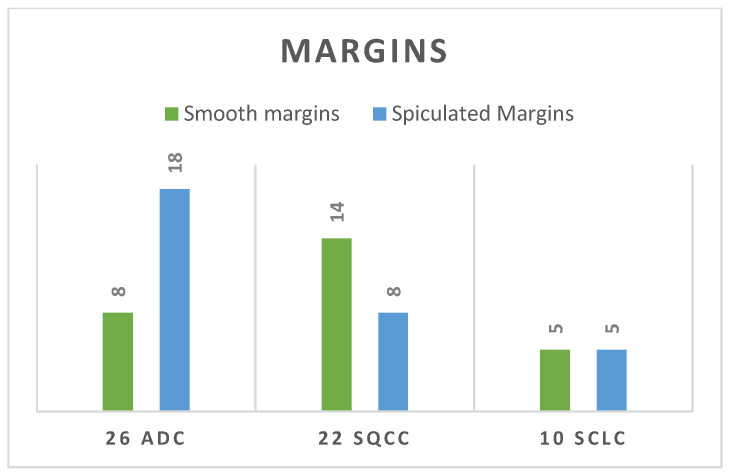
Tumoral margin distribution for each group.

In our study, a significantly higher proportion of cases in the ADC group had tumours with spiculated margins, with a percentage of 70% (18 cases), as seen in Figure 6. Regarding the SCLC group, there was an equal allocation of both spiculated and smooth margins. In contrast to these groups, 14 out of 22 cases with squamous cell carcinomas had smooth margins (63.6%), as seen in Figure 7.

#### 3.2.4. Cavitation

**Figure 8:**
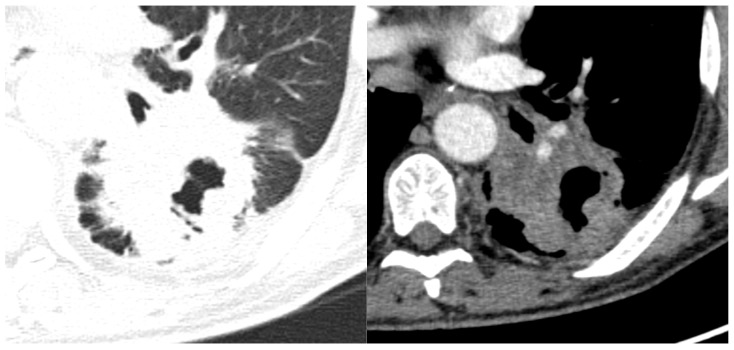
Axial CT of a peripheral SQCC with intratumoral cavitary lesion with anfractuous border; pleural invasion through spiculated processes which have no with the posterior costal arch can be observed.

**Figure 9:**
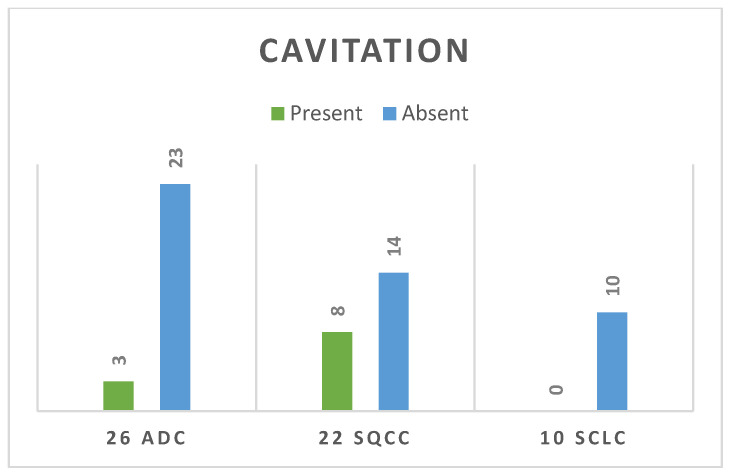
Presence or absence of cavitation feature.

Cavitation was defined as the presence of an air-containing space within the primary tumour that was not identifiable as an airway. Internal cavitations were noted in 36.3% of SQCC (Figure 8) cases, compared with the other groups with a *p* value less than 0.05 (*p* = 0.02). Only three cases were cavitary in the ADC group and none of the SCLC cases presented with cavitation, as shown in Figure 9.

#### 3.2.5. Local Invasion

**Figure 10:**
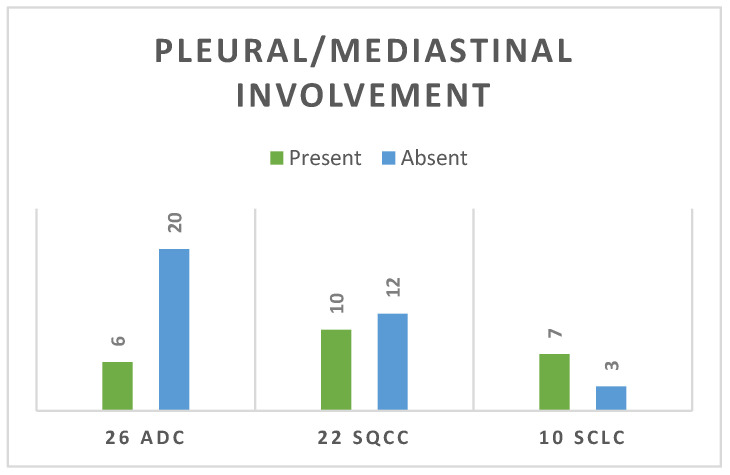
Presence or absence of locoregional (pleural or mediastinal) involvement.

Regarding local invasion, mediastinal and pleural invasion was considered when the tumour tissue had macroscopic involvement of the mediastinal fat or within the pleural cavity, while probable invasion represented by simple contact with either the mediastinum or pleura was not considered clear invasion in the statistical analyses nor in the staging. Only 23% of the ADC cases had pleural or mediastinal invasion; there was no significant percentage in the SQCC group (approximately 45%) (*p* = 0.02) while 70% of the SCLC group presented with pleural or mediastinal involvement (7 out of 10 cases), as it is shown in Figure 10.

#### 3.2.6. Other Features—Bronchial Obstruction and Vascular Bundle Thickening

No significant statistical difference was noted regarding bronchial obstruction or vascular bundle-thickening within the ADC and SCLC groups. Approximately 70% of the SQCC group had bronchial obstruction or vascular bundle-thickening, as can be seen in Table 2.

#### 3.2.7. Lymph Node Involvement

**Figure 11:**
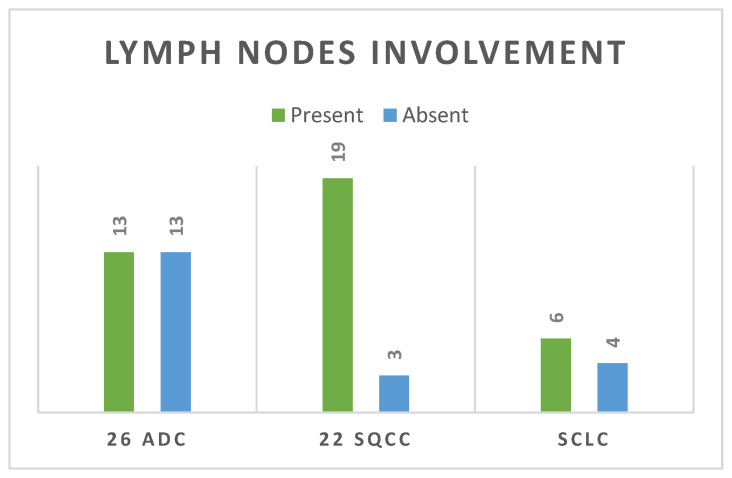
Lymph node involvement distribution.

**Figure 12:**
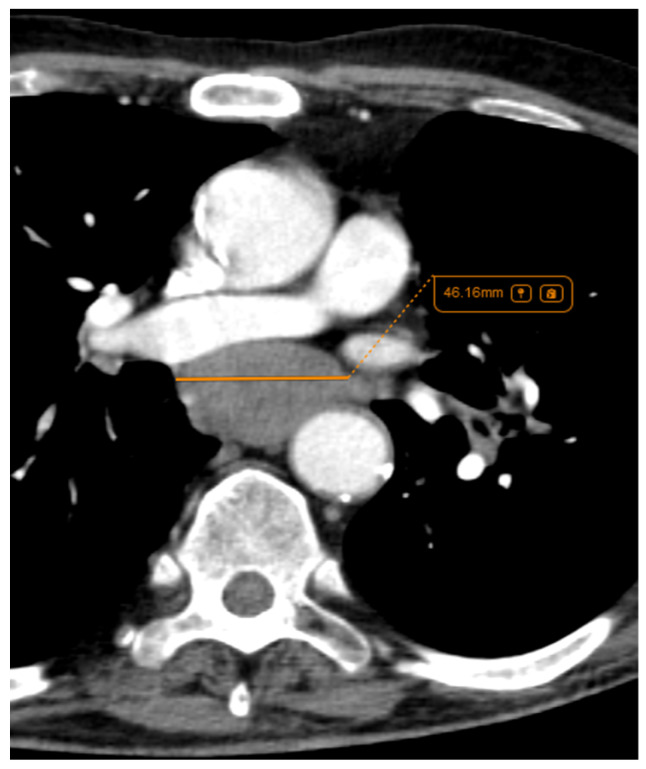
Axial CT—soft tissue view of a lymph node localised in the infracarinal region, from a left peripheral ADC.

**Figure 13:**
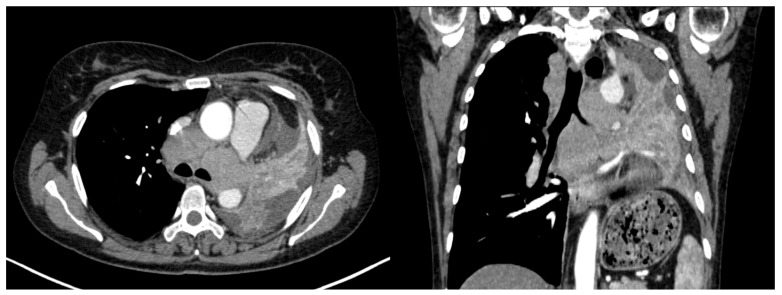
Axial and coronal CT of a left central SQCC with multiple mediastinal lymph nodes involving all mediastinal lymph stations.

Regarding lymph node involvement, there was an equal distribution in the ADC group (50%), while in the SQCC group there was a significantly higher proportion, with 19 cases (87%) presenting with lymph node involvement (Figure 11) with *p* = 0.02. In the current study, staging was conducted using the criteria of the Eighth Edition Lung Cancer Stage Classification [30]. Some examples of lymph node involvement can be found in Figure 12 and Figure 13.

## 4. Discussion

It is a known fact that even though female breast cancer has surpassed lung cancer (11.4%) as the most diagnosed cancer, with an estimated 2.3 million new cases (11.7%), lung cancer remains the leading cause of cancer death, with an estimated 1.8 million deaths (18%) [1,2]. Precise evaluation through imaging of the tumoral features and extent of the disease holds significance when determining the most effective treatment strategy. For radiologists to actively contribute to the comprehensive care of lung cancer patients, it is essential that they understand the ramifications of radiological observations on different tumoral descriptors and subsequent treatment choices.

Histopathological examination stands as the definitive method for diagnosing lung cancer and delineates the specific types of cancer [31]. It is essential to distinguish between lung malignancies, as targeted treatment modalities, such as surgical resection, chemotherapy, radiotherapy, and targeted therapy, can restrict disease progression and enhance survival outcomes for patients [32].

In this study, ADC was the most common subtype encountered, followed by SQCC, accounting for 26 cases and 22 cases, respectively, the distinction between them being critical for selecting the optimal treatment [33]. According to the literature, SQCC was the most common NSCLC subtype; however, in recent years, incidences of ADC increased and surpassed SQCC as the most common subtype of bronchial carcinoma [34,35]. In the last decade, more studies have shown increases in incidences of ADC. For example, Zhang et al. [36], in their study based on the data of 658 primary lung cancer patients, found that ADC was the most common subtype, followed by SQCC, then SCC, with the proportion of ADC increasing from 25.93 (1995–1997) to 56.36% (2013–2015). Our study can support these findings, which affects the evolution and treatment of the disease, considering that many cases with this subtype of NSCLC demonstrate positive results for targetable driver mutations such as epidermal growth factor receptor (EGFR), anaplastic lymphoma kinase (ALK), BRAF, and ROS1. Over the past few years, receptor tyrosine kinase inhibitors targeting these mutations, along with immunotherapies, such as programmed cell death protein 1 (PD-1) and cytotoxic T-lymphocyte-associated protein 4 (CTLA-4) inhibitors, have either replaced or complemented chemotherapy in eligible patients [37]. In this sense, increases in survival are likely due to earlier detection as well as improvements in treatment modalities, with the introduction of targeted and immune therapies.

Among the current study population, 32 lesions had a central location (55%), in which 15 were SQCC, 9 were SCLC, and 8 were ADC. It is important to note that 9 cases of SCLC presented central locations out of a total of 10 cases (90%). At the opposite end, ADC presented with a prevalent peripheral distribution (69%). Other studies showed that adenocarcinoma tends to develop in the peripheral regions of the lung [38], while incidences of central SQCC are well-known in many studies, including one by Mizushima et al. [39], who found over 235 squamous cell carcinomas from which 129 were peripheral and 106 were central. William Krimsky et al. found a total of 56 patients diagnosed with SCLC, of which 55% (*n* = 31) had peripheral and 45% (*n* = 25) had central SCLC [40]. Studies from radiology, oncology, and surgery indicate that the primary site plays a crucial role in predicting outcomes for metastatic lung tumours. Recognizing these prognostic factors serves as a valuable tool for guiding clinical treatment decisions. The primary site of the lung tumour holds prognostic significance, implying that patients with peripheral-type and central-type lung cancer exhibit distinct prognoses. Several studies have suggested that peripheral lung tumours offer a more favourable prognosis, regardless of whether they are squamous cell carcinomas or adenocarcinomas [41,42,43].

Internal cavitation was observed in 11 cases within the current study population, comprising 8 cases of squamous cell carcinoma and 3 cases of adenocarcinoma. Similar findings were reported by Chaudhuri et al., who, in their study of 100 cavitary lesions, identified 82 as SQCC, 11 as undifferentiated carcinomas of large polygonal-cell type, and 7 as ADC [44]. Regarding this study, all the SCLC group had solid structures. Data from the literature shows that, in contrast to noncavitary lung cancer, cavity formation appears to be more common in male patients and individuals with larger tumour sizes or squamous cell histology, correlating with a poorer prognosis. More EGFR mutations have been reported in noncavitary lung ADC, with a mutation rate of 47.3% and 33.6%, respectively [45], providing more treatment modalities, which in turn gives a better prognosis. Lan et al. 2021 [42] revealed a significantly shorter survival duration in patients with cavitary adenocarcinoma (*p* < 0.001), with a 5-year survival rate of 67.7% and a median survival time of 61.2 months. Patients without cavitary formation had a 5-year survival rate of 80.8%. Multivariate analysis revealed that cavity formation was an independent prognostic factor in adenocarcinoma (*p* = 0.028).

Our analysis involved a comparison of other various characteristics of pulmonary masses. Distinct features were noted among the three groups, encompassing the surface of tumour lobes, vascular bundle-thickening, and pleural involvement, aligning with the cancer’s pathological traits.

As in this study, adenocarcinoma typically exhibits histologic growth patterns characterized by acinar, papillary, and solid structures [46]. Consequently, the surface of adenocarcinoma lobes often presents with small, differentiated leaves and spinous processes, as is shown in our analysis, with 18 out of 26 cases of ADC presenting with spiculated margins. In contrast, squamous cell carcinoma tends to invade in tufts of tumour cells, resulting in relatively smooth surfaces [47], with 14 out of 22 cases revealing this feature in our study. Compared with other studies, where most cases of SCLC manifested as a lobulated mass rather than a spiculated mass, as was developed, for example, by Lee et al. [48], our study showed that there was an equal allocation of both spiculated and smooth margins in this group of lung cancers.

The vascular bundle sign, indicative of internal tissue fibrosis traction on adjacent structures, is more frequently associated with adenocarcinoma [49,50]. This disparity in the vascular bundle sign aligns with previous findings, such as those found by Kuriyama et al., who observed a higher prevalence of this sign in adenocarcinoma compared to small cell lung cancer [51]. Our analysis did not show a significant prevalence of this feature among all the cases included in the study.

Lymph node metastasis plays a key role in the treatment response and prognosis of SQCC patients [52]. We found out that the SQCC group had a significantly higher proportion of lymph node involvement, with 19 cases (87%) out of 22. In one study, Lou et al. 2020 showed that SQCC is more likely to have lymph node metastasis compared with adenocarcinoma [53] while Dong et al. 2019 indicated, in a retrospective study of 170 lung SQCC patients who underwent surgical treatment, that the presence of lymph node metastasis significantly influences the treatment response and prognosis. Significant differences were observed in both mean and median survival times between the groups with lymph node metastasis and without lymph node metastasis (*p* = 0.031). Specifically, the mean and median survival times were notably shorter in the lymph node metastasis group compared to the group without lymph node involvement. Furthermore, among individuals in the early T stages, those under 65 years old exhibit a higher inclination toward lymphatic metastasis. For young patients who are clinically lymph node-negative, prioritizing surgery and systemic lymphadenectomy over selective lymph node dissection may be considered as the primary therapeutic approach [52].

## 5. Conclusions

In clinical practice, histopathology serves as the gold standard for qualitatively diagnosing patients with lung tumours. However, some individuals, unable to tolerate invasive methods, may lack histopathological results. This study builds a correlation between CT features of lung cancer and histopathology, thereby providing a possible checklist for preliminary diagnosis, including CT characteristics such as morphological subtype predominance, location, margins, cavitation, or lymph node metastasis.

All these characteristics can provide information about the progression and prognosis of the patient, considering that NSCLCs are more frequent but tend to demonstrate positive results for targetable driver mutations, such as EGFR, increasing the overall survival. At the time of diagnosis, SCLC presents with distant spreads, which limits the opportunity to investigate the evolution of tumorigenesis and gene alterations at early stages but can have a rapidly positively response to chemotherapy. Patients with peripheral-type and central-type lung cancer exhibit distinct predictions, with several studies suggesting that peripheral lung tumours offer a more favourable prognosis. Cavity formation appears to be more common in male patients and individuals with larger tumour sizes or squamous cell histology, correlating with a poorer prognosis.

In this study the most common types were SQCC and ADC subtypes. CT showed that SQCC and SCLC subtypes tend to be centrally located, with internal cavitations being common in SQCC cases. In contrast, ADC tends to manifest peripherally and in a solid form.

While advocating for a comprehensive pathologic and molecular diagnosis in advanced lung cancer cases, both radiologists and clinicians should be familiar with the radiological features of different lung cancer subtypes. CT scans, being cost-effective, non-invasive, and readily available, serve as a diagnostic tool.

Due to the limited sample size in our study, particularly the small number of SCLC cases, further research with more SCLC cases is necessary. Using CT scans for a quick preliminary diagnosis can potentially reduce the waiting time for treatment, providing clinicians and patients with valuable information and allowing sufficient time for preparation for future treatments by clinicians, patients, and their families.

## Figures and Tables

**Table 1:** Age group distribution.

Age Group	ADC	SQCC	SCLC
40–50	1	1	3
51–60	1	4	1
61–70	12	11	4
71–80	10	6	2
>80	2	-	-

**Table 2:** Bronchial obstruction and vascular bundle thickening distribution.

Group	Bronchial Obstruction	Vascular Bundle-Thickening
ADC (26 total)	11	14
SQCC (22 total)	15	16
SCLC (10 total)	6	5

## Data Availability

The data presented in this study are available on request from the corresponding author. The data are not publicly available due to privacy.
